# Extensive transcriptome analysis correlates the plasticity of *Entamoeba histolytica* pathogenesis to rapid phenotype changes depending on the environment

**DOI:** 10.1038/srep35852

**Published:** 2016-10-21

**Authors:** Christian Weber, Mikael Koutero, Marie-Agnes Dillies, Hugo Varet, Cesar Lopez-Camarillo, Jean Yves Coppée, Chung-Chau Hon, Nancy Guillén

**Affiliations:** 1 Institut Pasteur, Cell Biology of Parasitism Unit, F-75015 Paris, France; 2 Inserm, U786, F-75015 Paris, France; 3 Institut Pasteur, Transcriptome and EpiGenome, BioMics, Center for Innovation and Technological Research, F-75015, Paris, France; 4 Institut Pasteur, Hub Bioinformatique et Biostatistique – Centre de Bioinformatique, Biostatistique et Biologie Intégrative (C3BI, USR 3756 IP CNRS) – F-75015 Paris, France; 5 Universidad Autonoma de la Ciudad de Mexico, Genomics Sciences Program, Mexico City, Mexico

## Abstract

Amoebiasis is a human infectious disease due to the amoeba parasite *Entamoeba histolytica*. The disease appears in only 20% of the infections. Diversity in phenotypes may occur within the same infectious strain in the gut; for instance, parasites can be commensal (in the intestinal lumen) or pathogenic (inside the tissue). The degree of pathogenesis of clinical isolates varies greatly. These findings raise the hypothesis that genetic derivation may account for amoebic diverse phenotypes. The main goal of this study was to analyse gene expression changes of a single virulent amoebic strain in different environmental contexts where it exhibit different degrees of virulence, namely isolated from humans and maintained through animal liver passages, in contact with the human colon and short or prolonged *in vitro* culture. The study reveals major transcriptome changes in virulent parasites upon contact with human colon explants, including genes related to sugar metabolism, cytoskeleton rearrangement, stress responses and DNA repair. Furthermore, in long-term cultured parasites, drastic changes in gene expression for proteins with functions for proteasome and tRNA activities were found. Globally we conclude that rapid changes in gene expression rather than genetic derivation can sustain the invasive phenotype of a single virulent isolate of *E. histolytica*.

The parasite *Entamoeba histolytica* is the etiological agent of amoebiasis, an infectious disease that affects the intestine and the liver of humans. Amoebiasis occurs at high incidence in large populations with limited modern sanitation systems. The infestation arises after ingestion of cysts contaminating water and food. Upon de-cystation, a vegetative cell, the trophozoite, is formed that colonizes the intestine or becomes invasive. During intestinal invasion, *E. histolytica* degrades the colonic mucosa and then interacts with the intestinal epithelium, crosses the basal lamina and disrupts the extracellular matrix (ECM). Trophozoites colonize the human gut mainly as commensals that feed on bacteria, these are able to invade the mucosa then initiating the acute phase of the disease. Intestinal amoebiasis has multiple clinical manifestations ranging from frequent mild diarrhoea to dysentery with blood and mucus in the stool. A major research interest on *E. histolytica* is driven by the observation that only one out of five infected persons develops the disease^1^ while most reported infections are asymptomatic. The development of the disease has been associated with human factors^2^, environmental conditions^3^,^4^ and especially with trophozoite virulence^5^. The amoebic invasive process can then induce an inflammatory response accompanied by disease-relevant cell death (for a review^6^). In some cases, subsequent to intestinal infection, or by direct acquisition through the blood, the parasite disseminates via the circulatory system, reaches the liver and induces hepatic abscesses that can be fatal for the infected person. Circulating trophozoites are confronted with the host’s innate immune response, in particular with cytotoxic compounds, as well as with oxygen tensions higher than those usually found in the gut. In addition, trophozoites are exposed to other host defence mechanisms – they are notably attacked by reactive oxygen and nitrogen intermediates produced by phagocyte cells, these compounds are highly concentrated in the infected tissue. For example, it has been shown experimentally that trophozoites invading the liver are highly sensitive to blood complement and tissue responses^7^,^8^. The diversity of human compartments that *E. histolytica* crosses during the infection combined with the variability of symptoms induced, suggest that this parasite expresses distinct transcriptional programs although the key components regulating gene expression remain to be described.

Following the sequencing and annotation of the *E. histolytica* genome^9^, microarray approaches have been developed to analyse gene expression in this organism. The data obtained revealed important regulatory networks involved in strain phenotype differences^10^,^11^,^12^, colonic and hepatic invasion^12^,^13^,^14^,^15^, responses to stress^16^,^17^, drug treatments^18^,^19^, metabolic changes^20^,^21^,^22^,^23^ and the encystation process^24^. Overall, these studies have identified diverse pathogenic factors, new pathways for drug treatment and some elements of transcriptional gene regulation. Thus, transcriptome approaches opened solid avenues to investigate the mechanism supporting invasion and pathogenicity of *E. histolytica*. Since this parasite only targets humans and the gut is the main reservoir for the cyst form, it is difficult to experimentally study infections in the context of amoebic human natural host aiming to understand the molecular basis of amoebiasis. Human colon explants have recently been used as an alternative system, and experiments have provided new knowledge to understand the pathophysiological bases of intestinal invasion^14^,^25^,^26^,^27^. For instance, the role of amoebic proteinases and human metalloproteinases^27^ in the degradation of the ECM and activation of immune responses has been outlined. The comparison of the gene expression profiles of parasites isolated from an asymptomatic carrier (i.e. the non-virulent Rahman strain) and from a symptomatic patient (i.e. the virulent HM1:IMSS strain) in contact with human colon explants has provided a global view of gene expression changes^11^. One remarkable feature of the virulent phenotype resides in the up-regulation of genes implicated in carbohydrate metabolism and processing of glycosylated residues of compounds building the intestinal barrier. However, it can be argued that trophozoites from the isolates used in these experiments have been in culture for decades and may harbour some genetic deviations influencing their virulence. In conjunction with the expression of genes encoding the so-called virulence factors, the infectious process also requires the expression of genes whose products are important for adaptation to different host responses and to counteract environmental factors of the invaded tissues.

In this work we hypothesized that for a given *E. histolytica* strain, changes in the environment (in particular those occurring during the interaction with the human host) trigger the major gene expression marks related to different degrees of virulence. To test this hypothesis, we applied high-throughput RNA sequencing to profile, for the first time, samples from the same isolate (HM1:IMSS) in contact with different environments. We harvested trophozoites from liver abscess experimentally induced in the hamster animal model, a condition enhancing amoebic virulence, and from these virulent parasites loaded in contact with human colon explants, reflecting the intestinal invasive process. Searching for factors that might be implicated in the aggravation of the disease, we first defined a set of genes specifically expressed in virulent trophozoites isolated from the liver that we further refined by studying trophozoites interacting with the human colon. In addition to stress responses and DNA repair, the expression of important cytoskeleton-related factors was the mark of cultured virulent parasites. Remarkably, virulent parasites change their profile upon interaction with the human colon by increasing the expression of genes related to metabolism of carbohydrates and glycosylated residues. The profiles established for virulent trophozoites were compared to those of long-term *in vitro* cultured parasites of the same strain that have acquired a virulence-attenuated phenotype^7^. The marks associated with virulence and intestinal invasion are lost in virulence-attenuated amoebae, which appeared highly active in proteasome activities and repressed in tRNA transfer enzymes. Based on our results we concluded that rapid and highly specific changes in the amoebic transcriptome, rather than genetic variation of the amoeba isolate, can account for the invasion of human colon by virulent *E. histolytica*.

## Results and Discussion

### Characterization of the transcriptome of *Entamoeba histolytica* with different degrees of virulence

**Figure 1:**
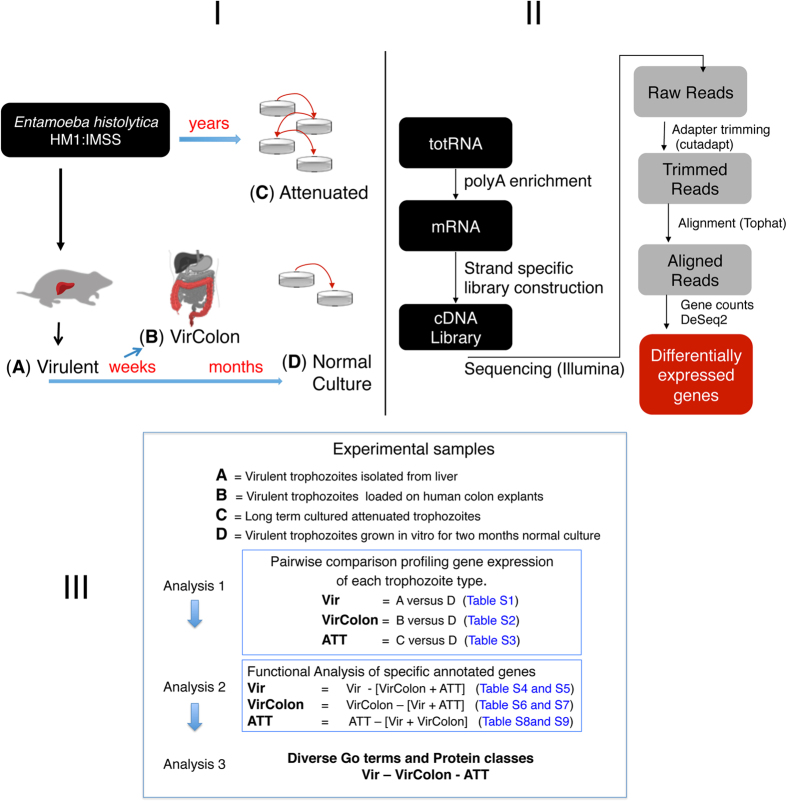
Workflow for the experimental procedures. I. The amoeba strain HM1-IMSS was isolated form the liver of hamster and then cultivated in several conditions: (**A**) *in vitro* overnight (Vir condition), (**B**) *in vitro* for two weeks and then deposited on the top of human colon explants (Vir Colon condition) or (**D**) *in vitro* for several weeks (Normal condition). Long-term cultured parasites ATT were also tested (**C**). II. RNAs were organically isolated, polyA-enriched and directional libraries were constructed and sequenced. The raw reads were filtered for quality and sequence contaminants and aligned to the reference genome (TopHat) to finally compute differential expression levels (DeSeq2). III. Paths followed for the bioinformatics analysis of the data obtained by RNASeq.

To analyse transcriptional changes in trophozoites of the same strain undergoing changes in virulence we used RNAseq. The RNA libraries were constructed from polyA + mRNA from the HM1:IMSS strain grown in four different conditions (Fig. 1A) Virulent parasites extracted from liver abscesses of infected hamsters (Vir), B) Virulent parasites exposed to explants of human colon (VirColon), C) long-term cultured virulence-attenuated amoeba (ATT) that have lost the ability to form liver abscesses and D) trophozoites short-time cultured (Normal Culture) for roughly lest than three months (24 passages). Three replicates of each condition were performed resulting in 12 RNA libraries in total that were sequenced on an Illumina HiSeq 2000 platform (see Material and Methods). Data were processed, using bioinformatical and statistical workflows as already published^28^. Coding gene models were based on the bona fide gene models defined in previous work (n = 7312)^28^. We determined that in all the diverse conditions almost 95% of the annotated coding regions had at least one read indicating that our dataset is deep enough to analyse the majority of annotated transcripts. Differentially expressed genes were defined as genes with ≤5% false discovery rate and ≥2-fold or ≤0.5-fold changed expression rates following the statistical analysis performed with DESeq2 as described in the Materials and Methods section (Supplementary Tables S1 to S3).

### Pairwise comparison of gene expression profiles

**Figure 2:**
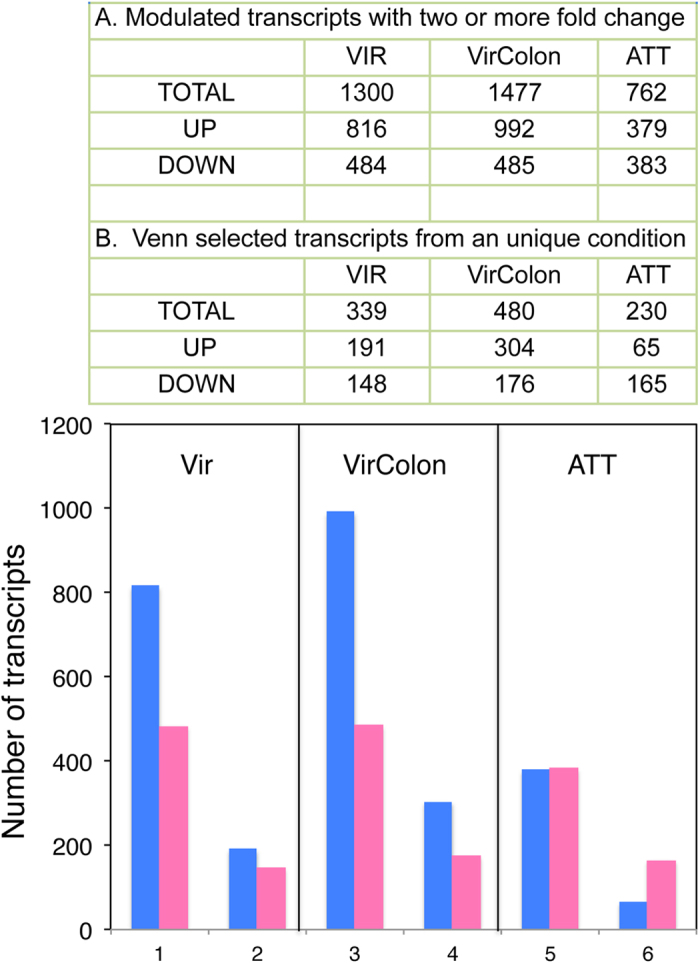
Analysis of the differentially expressed genes. After mapping of short reads on the amoebic genome, the number of modulated genes was determined for each condition (**A**) in comparison to genes from the normal culture condition. Comparing only the annotated genes in a Venn diagram the number of gene modulated unique to each condition (**B**) was determined. The figure in the bottom summarizes the different values: those represented in bleu indicating the UP regulated genes and those in pink correspond to down regulated one. Two bars appears per condition and these correspond firstly (left bar) to all transcript modulated by condition and secondly (right bar), to unique transcripts modulated in each condition.

To characterize the differences in gene expression of the HM1:IMSS strain in the four conditions tested; we used the virulent parasites grown for a limited period in culture medium (referred as D) as the reference. Pairwise comparisons were performed with the three growth conditions, A, B and C. (Fig. 1 and Supplementary Table S2). The comparative analysis identified the differentially expressed genes (Fig. 2), with 1300 genes in the condition A (Vir) (816 up- and 484 down-regulated); 1477 genes (992 up- and 485 down-regulated) in condition B (VirColon) and 762 genes (379 up- and 383 down-regulated) in condition C (ATT) with respect to the reference condition (D).

### Expression profile of virulent parasites isolated from the liver

The A versus D comparison highlights genes that are modulated upon extraction of parasite from the liver tissue and overnight culture. Among the 816 up-regulated genes, 381 had been previously annotated whereas 435 were of unknown function. The data (Supplementary Tables S4 to S9) showed important changes in genes related to virulence, and in particular genes involved in stress responses, as previously suggested from microarray data^14^,^15^,^16^. Genes encoding several so-called heat shock proteins were up-regulated, as well as genes for proteasome and protein degradation pathway components described as over expressed in stress conditions. We observed for the first time important changes affecting genes related to the cytoskeleton. Among the 214 annotated down-regulated genes (Supplementary Table S5), several members of the AIG family (GTPase immunity-associated protein family) encoding genes are the most differentially expressed.

### Expression profile of virulent parasites interacting with human colon explants

For virulent trophozoites in contact with human colon explants, a well-defined gene expression pattern indicated the ability of *E. histolytica* to trigger rapid expression changes during early stages of colon invasion (comparison B versus D). Among the 994, up-regulated genes, 464 were already annotated and included genes involved in carbohydrate metabolism and protein glycosylation, AIG factors, the Gal-GalNAc lectin (heavy and intermediate subunits) and competence EC (Supplementary Table S6). We compared the file corresponding to annotated up-regulated genes in contact with the human colon with our previously published data (Supplementary Table S10) obtained by microarray experiments^12^ and confirmed that incubation with the colon triggers a unique gene expression profile that is qualitatively different from the one of parasites from the same isolate freshly isolated from the liver (B/D versus A/D), or from long-term cultured parasites (B/D versus C/D). In particular, regulators of nonsense transcripts and alpha- or beta-amylase encoding genes were found, the latter being an essential protein in the pathogenic process of *E. histolytica*^12^. Compared to microarrays, RNASeq appears highly sensitive allowing for a deeper analysis of the stress response (see below). Among the 485 down-regulated genes in the B vs. D comparison (Supplementary Table S7), the 228 previously annotated genes provided evidence that the transcription of some genes involved in amoeba pathogenicity was diminished when amoebae were in contact with the human colon, notably the genes coding for the Gal-GalNAc lectin light chains and the lysine- and glutamic acid-rich protein 1 (KERP1).

### Expression profile of virulence-attenuated parasites

Attenuated parasites (ATT) were derived from the virulent HM1:IMSS strain by prolonged axenic culture^7^. Among the 379 up-regulated genes (151 annotated) we identified (Supplementary Table S8) several genes belonging to the *ariel* multicopy gene family which is specific to *E. histolytica* species among the *Entamoeba* genus. Although this family was identified 15 years ago, no function has been attributed to the ARIEL protein so far^29^. Genes encoding Hsp20 -Hsp70 or START domain-containing proteins were up-regulated. All these gene categories have been previously identified by microarray experiments as being up regulated in strains with low virulence, such as the Rahman strain^12^. Among the 212 annotated and down-regulated genes of ATT trophozoites (Supplementary Table S9), we identified genes encoding proteins associated with the parasite surface, including the immuno-dominant variable antigen M17^30^, the BspA gene family^31^ or the serine-threonine-isoleucine-rich protein^32^ as well as genes encoding numerous signalling molecules such as small GTPAses and protein kinases^33^.

### Functional analysis of modulated genes

The PANTHER classification system, searching for GO terms, allowed the functional interpretation of RNASeq datasets (Up or Down genes)^34^. The *E. histolytica* genome was only recently added to the PANTHER database and it is important to note that not all genome data entries are associated with a hit in PANTHER protein class, family or pathway classifications, due to the incomplete genome annotation.

**Figure 3:**
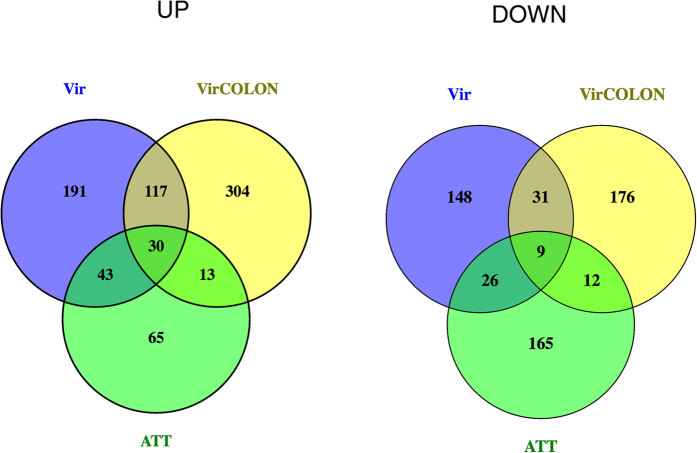
Venn diagram of the differentially expressed genes. The number in each circle represents the amount of differentially expressed genes between the different comparisons (test versus control). Only the annotated genes were considered. The overlapping number stands for the mutual differentially expressed genes between the different comparisons and the non overlapping numbers specify the genes unique to each condition: virulent (Vir), Virulent on the colon (Vir Colon) and long term cultured ATT (attenuated). Up and Down regulated genes were determined.

For functional predictions, we first used Venn diagrams to identify genes whose expression regulation was specific to one of the three conditions, as well as genes whose expression regulation was observed in two or three conditions (Fig. 3). The latter were removed and were not further analysed. We found 304, 191 and 65 genes that were up-regulated in conditions B (VirColon, amoebae in contact with colon), A (Vir, virulent amoebae from liver) and C (ATT, long-term cultured, virulence-attenuated amoebae) respectively. Down-regulated genes were analysed similarly revealing 176, 148 and 165 genes specifically repressed in VirColon, Vir and ATT respectively.

**Figure 4:**
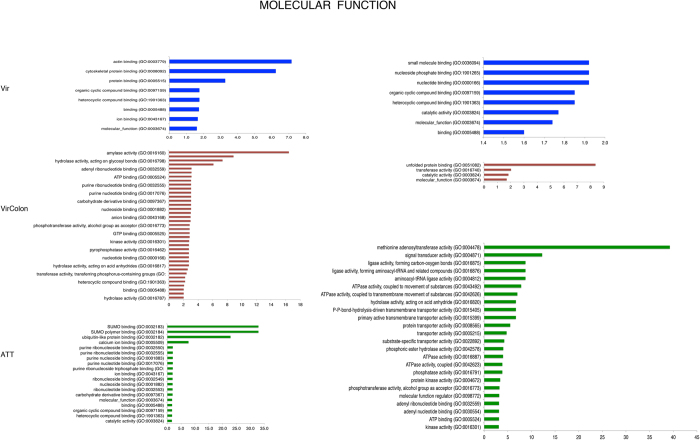
Distribution of GO functional classifications. Upon analysis with Panther tools^34^, the distribution of the fold enrichment levels of terms for diverse molecular functions categories for Vir, VirColon and ATT (Up and Down modulated genes) is represented.

**Figure 5:**
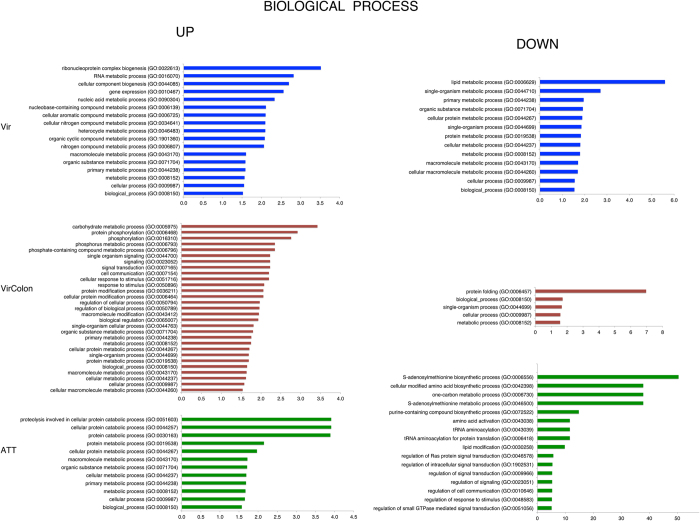
Distribution of GO functional classifications. Upon analysis with Panther tools^34^, the distribution of the fold enrichment levels of terms for diverse biological process categories for Vir, VirColon and ATT (Up and Down modulated genes) is represented.

Following the Venn selections, an enrichment analysis was performed to identify GO categories that were over- or under-represented among the list of the specific differentially expressed genes. GO terms were sorted into the different subcategories for molecular function (MF) (Fig. 4) and biological processes (BP) (Fig. 5). Diverse levels of enrichment were found in the two main GO aspects (MF, BP) for the three datasets (A/D, B/D and C/D). For the induced genes (Table 1), the Vir condition (191 genes) showed a significant enrichment in MF for actin binding (GO:0003779) and cytoskeletal protein binding (GO:0008092) categories with 7 and 6 fold enrichments respectively. In BP we found the ribonucleoprotein complex biogenesis (GO:0022613) and with 4 fold enrichment, RNA metabolic process (GO:0016070) with 2.82 fold, while 65 genes were not classified.

In MF the GO terms analysis for up-regulated genes in the VirColon condition (304 genes) highlighted amylase activity (GO:0016160) with 16 fold enrichment, hydrolase activity hydrolysing O-glycosyl compounds (GO:0004553) with 9 fold, and hydrolase activity acting on glycosyl bonds (GO:0016798) with 7 fold enrichment, while 65 genes were not classified. For the biological processes, carbohydrate metabolic process (GO:0005975) was enriched 3 fold, while 83 genes were not classified.

Finally, the GO terms analysis for MF in the ATT condition revealed the SUMO binding (GO:0032183), SUMO polymer binding (GO:0032184) with both 33 fold enrichment and the ubiquitin-like protein binding (GO:0032182) with 23 fold enrichment. The BP found as the most significant were the cellular protein catabolic process (GO:0044257), proteolysis involved in cellular protein catabolic process (GO:0051603) and protein catabolic process (GO:0030163) all with 4 fold enrichment. SUMO is the Small Ubiquitin-related Modifier protein that is important for gene transcription regulation^35^. SUMO modifies many proteins including promoter-specific transcription factors, cofactors and chromatin-modifying enzymes. A total of 14 genes were unclassified.

Concerning the GO term analysis of down-regulated genes (Figs 4 and 5) in the Vir condition, lipid metabolism (GO:0006629) was the most relevant with 6 fold enrichment whereas 47 genes were unclassified.

The most relevant GO term found for genes down regulated in the VirColon condition in BP was the protein-folding category (GO:0006457) with 7 fold. In MF the unfolded protein binding (GO:0051082) was enriched 8 fold. 43 genes were unclassified.

Finally numerous GO term enrichments were identified in the ATT condition indicating a high impact in down-regulation of genes for this amoebic strain. In BP the following categories were significantly enriched: S-adenosylmethionine biosynthetic process (GO:0006556) with 35 fold, purine-containing compound biosynthetic process (GO:0072522) with 14 fold enrichment, tRNA aminoacylation for protein translation (GO:0006418) with 9 fold enrichment, tRNA aminoacylation (GO:0043039) with 9 fold enrichment, amino acid activation (GO:0043038) with 9, fold enrichment lipid modification (GO:0030258) with 9 fold enrichment and regulation of Ras protein signal transduction (GO:0046578) with 5 fold enrichment and several others with changes less than 5-fold. Only 38 genes were unclassified. Many molecular functions were associated with these GO terms (Figs 4 and 5): methionine adenosyltransferase activity (GO:0004478) with 39 fold, signal transducer activity (GO:0004871) with 12 fold and aminoacyl-tRNA ligase activity (GO:0004812), ligase activity, forming aminoacyl-tRNA and related compounds (GO:0016876), ligase activity, forming carbon-oxygen bonds (GO:0016875), all with 9 fold enrichment.

Overall the GO term analysis determined the high specificity of transcriptome patterns for each of the three conditions tested.

### Protein classes highly represented in the different growth conditions

**Figure 6:**
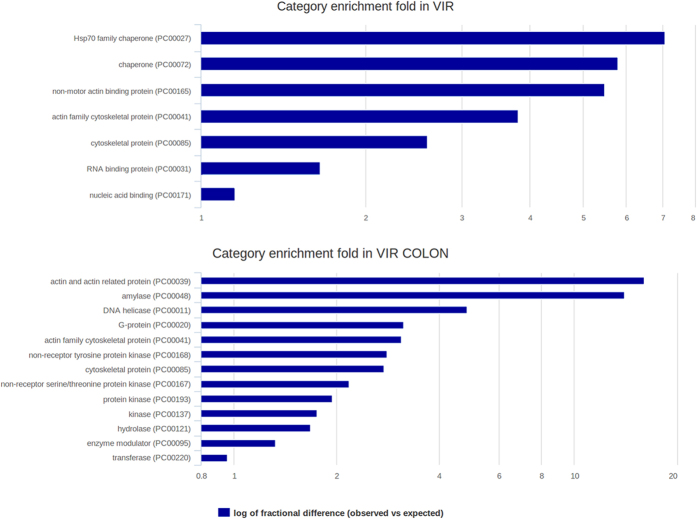
Enrichment of protein classes for Vir and VirColon UP regulated genes. The log of fractional difference between observed versus expected is plotted upon identification of protein classes representatives of Vir and Vir colon whose genes are up regulated.

To generate a more focused view of the GO terms, we looked at protein class enrichments between the PANTHER categories. The software plots the Log of fractional difference (observed versus expected), which is calculated for each testing list as: (number of genes for the category - number of genes expected)/number of genes expected (Fig. 6). For the genes up regulated in the Vir condition, we observed a significant enrichment for Heat Shock proteins where Hsp70A, Hsp70 and Hsp90 appearing as the most overrepresented (Fig. 6). For the first time we revealed the association of genes involved in RNA metabolism and amoebic virulence. This includes the DNA-RNA polymerase (EHI_122780, EHI_125350), mRNA splicing factors (EHI_049370, EHI_183900, EHI_060350, polyadenylation factors (EHI_067580)) as well as diverse components of the mRNA transduction machinery (Supplementary Table S11).

In the up-regulated genes the actin-rich cytoskeleton is clearly a mark for virulence in the context of liver isolated parasites, (Supplementary Table S12) showing over 32 proteins identified including actin, actin binders and cytoskeleton constituents as the most significant. These proteins belong to the actin-binding proteins category with filament crosslinking activities able to organize microfilaments in orthogonal and/or parallel networks^36^, including: filamin (EHI_104630, EHI_167130), villin (EHI_007480), gelsolin (EHI_009570), filopodin (EHI_080740), cortexillin (EHI_103430), Diaphanous-Formin (EHI_190990, EHI_192460, EHI_118260) Paxillin (EHI_069060, EHI_022960) as well as actin binders including coronin (EHI_083590, EHI_122800), Myosin II (EHI_110180), cofilin (EHI_186840) and a recently identified Arp related protein Arpv2 (EHI_048630) unique to *E. histolytica*^33^.

Amylases, DNA repair and primary metabolism are the mark for VirColon. Several alpha-amylases (EHI_153100, EHI_152880, EHI_023360, EHI_129810) and beta-amylase (EHI_153590, EHI_009020, EHI_049700, EHI_058340, EHI_148800, EHI_035700) appeared represented. Previously^12^ the beta-amylase EHI_192590 allele showed (in microarray experiments) a fold change up to 25 in the comparison between *E. histolytica* HM1:IMSS strain (virulent) and *E. histolytica* Rhaman strain (non-virulent) cultured *in vitro* or during their interaction with human colon explants. Here expression of the EHI_192590 allele didn’t show any change probably because we are comparing only an unique virulent strain in different conditions; instead significant overexpression of four copies of beta-amylase encoding gene was observed (Supplementary Table S13) strongly sustaining our conclusion on the role of beta-amylase in the infectious process.

Several genes encoding proteins with helicase activities involved in DNA repair and genome stability maintenance were up-regulated and overrepresented in trophozoites in contact with host colon (Supplementary Table S13). The DNA damage response represents a survival mechanism that confers cellular protection against stresses induced by DNA-damaging agents, several classes of antibiotics, and oxidative agents that may attack amoebae during colon invasion. Two genes potentially involved in nucleotide excision repair (NER) of DNA, namely ERCC2 (EHI_054240) and ERCC3 (EHI_088430), were significantly overexpressed. Both ERCC2 and ERCC3 are DNA helicase component of the core-TFIIH basal transcription factor involved in NER and RNA transcription by RNA polymerase II in human cells^37^. We also found an increased expression of a gene (EHI_187240) with high homology to WRNIP1, a Werner DNA helicase interacting protein 1, which functions as a modulator for initiation events during DNA polymerase delta-mediated DNA synthesis and sensor of DNA damage^38^. In addition, two genes homologous to genes coding for DNA replication licensing factors, MCM2 (EHI_117970) and MCM7 (EHI_158110), involved in DNA replication and cell division in higher eukaryotes^39^ were also up-regulated. Intriguingly, two genes (EHI_178520, EHI_035550) sharing homologies with regulator of nonsense transcripts 1 (UPF1) were up-regulated in virulent trophozoites. In human cells, UPF proteins have RNA-dependent helicase and ATPase activities required for nonsense-mediated decay (NMD) of mRNAs containing premature stop codons^40^. Notably, NMD triggered by mRNA-mRNA interactions have been linked to cell migration and invasion of cancer cells^41^.

**Figure 7:**
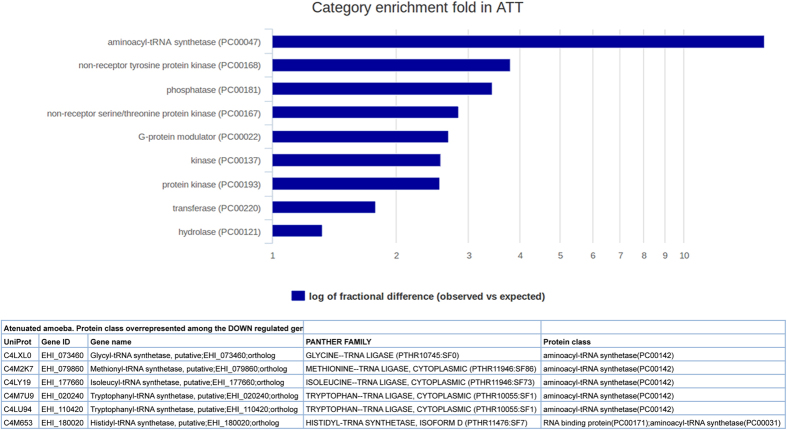
Enrichment of protein classes for ATT down regulated genes. The log of fractional difference between observed versus expected is plotted upon identification of protein classes representatives of ATT condition whose genes are down regulated.

The strikingly strong enrichment found among the up-regulated categories for ATT trophozoites concerned genes encoding E3 ubiquitin ligase (EHI_142060, EHI_158200, EHI_098590), a protein transferring ubiquitin to lysine residues of a protein to target it to the proteasome for degradation (Supplementary Table S14). The U-ligases found here contain a Zinc finger domain (RING domain). Ubiquitin hydrolases (EHI_064460), Ubiquitin conjugating enzyme (EHI_100110), several cysteine proteases and proteasome subunits were identified (Supplementary Table S14). Among the down-regulated genes for the conditions Vir and VirColon, no significant overrepresentation of protein classes was found. In contrast, for the ATT condition numerous signalling molecules including small GTPases (PC 00022), serine/tyrosine protein kinase (PC00167) and aminoacyl-tRNA synthetase (PC00047) with glycyl, methionyl, isoleucyl, tryptophanyl and histidyl tRNA synthetases were identified (Fig. 7). These are enzymes that link the appropriate amino acid onto its cognate tRNA to allow mRNA translation into proteins.

Finally, in order to try to identify common potential regulatory elements enhancing gene expression we performed a gene clustering analysis for the UP regulated genes. This confirmed the major findings above described for the diverse treatments applied to *E. hystolyica* HM1:IMSS (Supplementary Fig. 2). The data provide gene identities useful for further bioinformatics and experimental analysis of 5′ or 3′ elements important for gene expression regulation in each condition.

## General Conclusion

**Figure 8:**
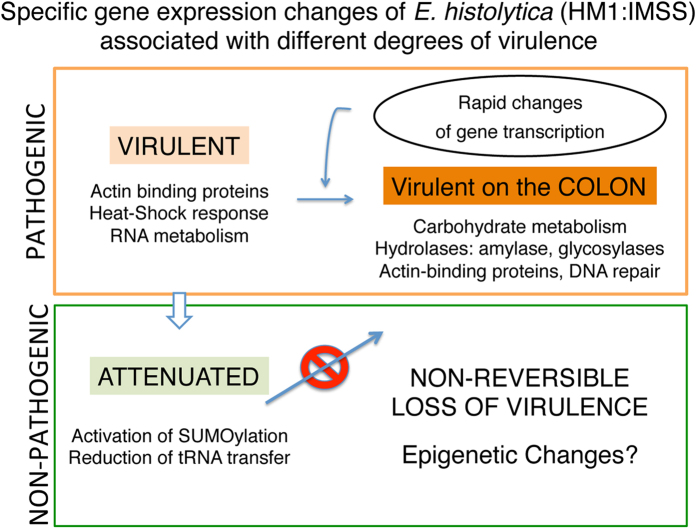
Summary and conclusions. *E. histolytica* HM1:IMSS virulent strain rapidly adapts its transcriptome according to changes of the environment. To trigger the pathogenic process, the parasite nourishes upon degradation of the mucus layer, overcomes the various tissue stresses and moves into the deep layers of the intestine, all these fuctions are rapidly activated upon contact with the intestine. Trophozoites adapted to long-term culture are not able to trigger these reactions and instead, protein degradation is highly active as well as the reduction of amino acids supply to the ribosome for protein synthesis.

In the present study, we used extensive comparative transcriptome analysis to determine the gene expression profiles of *E. histolytica* associated with virulence in the context of intestinal and liver infection. According to the read coverage obtained in the RNASeq approach we conclude that globally the entire genome appears to be transcribed in all tested conditions. The complete analysis of gene expression of highly virulent trophozoites freshly isolated from liver abscess and grown for only 16 hours *in vitro* identifies for the first time important features implicated in this phenotype including actin cytoskeleton, heat shock response and RNA metabolism. Changes in stress responses have already been observed in precedent work but the analysis presented here is clearly more exhaustive. The analysis of one amoebic strain in diverse environmental conditions influencing pathogenicity revealed that modulation of the amoebic transcriptome is capital for parasite adaptation allowing survival, growth and invasive behaviour. We thus discovered the genes that rapidly responded to the contact of this parasite with human colon. The patterns of differentially expressed genes of amoebae originating from the same strain and challenged with different environmental conditions closely correlates to the gene responses identified during the comparison of two particular isolates^12^: one from an asymptomatic carrier (*E. histolytica* Rhaman strain) and the other from a symptomatic patient (*E. histolytica* HM1:IMSS) and allows to conclude that only the contact of virulent trophozoites with the human colon triggers an unique gene transcription profile. The genes identified as significantly modulated in the process of human colon invasion confirm that metabolic processes including the activity of glycosylases, together with cytoskeleton and DNA repair activities, are the most important mechanisms underlying amoebic intestine invasion (Fig. 8). For the first time, we also highlighted two important phenotypic features of long-term cultured virulence-attenuated trophozoites, namely the activation of SUMO activity and the down-regulation of tRNA synthetases, suggesting that in these trophozoites the increase of proteasome activities and the down-regulation of the translational machinery may be involved in gene regulation, which is different from the virulent parasites.

The major conclusion of this work is that a rapid shift in gene expression is the most important phenomenon driving amoebic pathogenicity during its interaction with the human colon. In our working model, we consider several factors that may explain the shift of the intestinal amoebic phenotype. First, a change in the composition of the bacterial flora which should take place at any time. For instance, it is known that co-infection in the presence of some Enterobacteriaceae increases the virulence of the parasite^42^. Another plausible factor is the human immune response because the amoebae are chemoattracted by pro-inflammatory molecules such as tumor necrosis factor^43^ and some intestinal resident cells behaving like a TNF reservoir (eg. mast cells) potentially activated at an early stage of infection. In a general view, thank to the impact of this work, the hypothesis that genetic variability sustains differences in pathogenicity^44^ gain in precision and seems highly related to the lost of invasive ability of commensal parasites, a fact opening new avenues to investigate whether epigenetic mechanisms may sustain such commensal behaviour. Our findings provide the first insight into the molecular basis of gene expression variation of pathogenic *E. histolytica* according to the environmental conditions encountered during invasive infection.

## Material and Methods

### *In vitro* culture of *E. histolytica*

*Entamoeba histolytica* HM1:IMSS is a virulent strain isolated in 1967 from a colonic biopsy of rectal ulcer from adult human male with amoebic dysentery, Mexico City, Mexico. The HM1-IMSS was deposited in the American strain collection (ATCCH 30459TM) and it is a gift of Professor Ruy Perez Tamayo and Dr Alfonso Olivos (UNAM, Mexico). To maintain virulence, the HM1-IMSS strain has been passed periodically through the liver of hamsters (roughly 180 passages since isolation). The axenic *Entamoeba histolytica* strain HM1:IMSS was cultured in TYI-S-33 medium at 37 °C^45^. The fresh liver isolate was maintained in culture for 16 hours, for two weeks or maximally three months. Cultures at two months were used as the reference condition for the transcriptome comparisons. Attenuated trophozoites were derived from the HM1-IMSS virulent strain by prolonged axenic culture (more than 10 years) and have lost the ability to form liver abscesses in the hamster animal model^7^.

### Hepatic inoculation procedure

Four-weeks-old male Syrian golden hamsters (*Mesocricetus auratus*), with a weight ranging from 90 to 100 g, were inoculated by the intraportal route with 1 × 10^6^ virulent HM1:IMSS trophozoites. Treatment of animals and surgical procedures were carried out in accordance with relevant guidelines and regulation. The experimental protocole (N° ND/JLA-06.254) has been published^8^ and was approved by “Service Hygiène, securité et protection de l’environement” of Institut Pasteur, France. At 7 days post-infection, hamsters were sacrificed and livers removed. For RNASeq experiments, pieces of infected liver were incubated in TY1-S-33 medium and parasites were allowed to adhere to the culture tube for 16 hours. Liver tissue was then discarded and the pool of trophozoites used to purify RNA or for further growth.

### Interaction of *E. histolytica* with human colon explants

Previous experimental published conditions were used for handling human colon pieces^25^ and RNA purification^12^. Briefly, 1.6 × 10^5^ trophozoites were added to the luminal face of the colon and incubated in Krebs buffer at 37 °C for 1 and 7 hours. After 1 hour of incubation, trophozoites interacting with the mucus were collected by recovering the mucus layer for RNA purification. Tissues (3 independent samples) were processed in accordance with the French government’s guidelines for research on human tissues and the French Bioethics Act. Patient-written informed consent was obtained at Foch Hospital and the data were analysed anonymously at the Pasteur Institute. The applied protocol was approved by a regional investigational review board (Comité de Protection des Personnes, Ile de France VII, Paris, France) and an institutional review board (Institut Pasteur Recherche Biomedicale, Paris, France; reference RBM/2009.50).

### RNA extraction, library construction and sequencing

Total RNA was extracted from approximately 1 × 10^6^ *E. histolytica* trophozoites (with each culture performed in triplicates) of using Trizol (Invitrogen). The polyA fraction was purified from 10 μg of total RNA using Dynabeads according to the manufacturer’s instructions (Thermofisher). Libraries were constructed using ScriptSeq mRNA-Seq Library Preparation Kit (Illumina) following manufacturer’s recommendations and were quality controlled using Agilent Bioanalyzer. Sequencing was performed on a HiSeq 2000 (Illumina) to obtain 58 base single-end reads. Adapter sequences were trimmed using Cutadapt^46^ and reads shorter than 15 nucleotides removed.

These processed reads data are available in the ArrayExpress database (www.ebi.ac.uk/arrayexpress) under accession number E-MTAB-4882.

### Analysis of transcriptome sequencing data

Sequence reads were mapped to the *E. histolytica* genome assembly (AmoebaDB v1.7, http://amoebadb.org/amoeba/) using Tophat version 2.0.6 with default parameters. Count data for coding genes (based on the bona fide gene models defined in previous work^28^ were analysed using R version 3.2.2^47^ and the DESeq2 Bioconductor package version 1.10.0^48^. Data were normalized with DESeq2 and the default parameter. The dispersion estimation and statistical test for differential expression were performed with default parameters (including outlier detection and independent filtering). Raw p-values for each comparison (each growth condition versus control) were adjusted for multiple testing according to the Benjamini and Hochsberg (BH) procedure^49^ and genes with an adjusted p-value lower than 0.05 were considered differentially expressed. A Principal Component Analysis (PCA) was performed to explore the structure of the data (Supplementary Fig. 1). The input data for the PCA was a subset of the VST-transformed counts matrix containing the 500 most variant genes. GO terms analysis and Protein classes identification was performed with the tools of PANTHER^34^. Only gene expression rates with a fold-change greater than 2 or lower than 0.5 were considered. To draw Venn diagrams we used Venny (http://bioinfogp.cnb.csic.es/tools/venny/index.html). We used the lists of differentially expressed genes resulting from the comparison of virulent HM1:IMSS extracted from the liver, virulent HM1:IMSS incubated on human colon, attenuated HM1:IMSS versus HM1:IMSS in culture.

## Additional Information

**How to cite this article**: Weber, C. *et al*. Extensive transcriptome analysis correlates the plasticity of *Entamoeba histolytica* pathogenesis to rapid phenotype changes depending on the environment. *Sci. Rep.* **6**, 35852; doi: 10.1038/srep35852 (2016).

## Supplementary Material

Supplementary Information

Supplementary Table S1

Supplementary Table S2

Supplementary Table S3

Supplementary Table S4

Supplementary Table S5

Supplementary Table S6

Supplementary Table S7

Supplementary Table S8

Supplementary Table S9

Supplementary Table S10

Supplementary Table S11

Supplementary Table S12

Supplementary Table S13

Supplementary Table S14

## Figures and Tables

**Table 1:** GO terms for up regulated genes.

	Fold	P-Value
**Molecular function**		
Vir		
actin binding (GO:0003779)	7	7.81E-04
cytoskeletal protein binding (GO:0008092)	6	2.59E-03
protein binding (GO:0005515)	3	5.26E-04
Vir Colon		
hydrolase activity, hydrolyzing O-glycosyl compounds (GO:0004553)	9	3.18E-05
hydrolase activity, acting on glycosyl bonds (GO:0016798)	7	1.99E-04
GTPase activity (GO:0003924)	6	2.75E-02
ATP binding (GO:0005524)	3	9.05E-18
adenyl nucleotide binding (GO:0030554)	3	9.05E-18
adenyl ribonucleotide binding (GO:0032559)	3	9.05E-18
carbohydrate derivative binding (GO:0097367)	3	3.88E-26
purine ribonucleoside triphosphate binding (GO:0035639)	3	1.54E-25
purine nucleotide binding (GO:0017076)	3	1.54E-25
purine nucleoside binding (GO:0001883)	3	1.54E-25
purine ribonucleotide binding (GO:0032555)	3	1.54E-25
purine ribonucleoside binding (GO:0032550)	3	1.54E-25
nucleoside binding (GO:0001882)	3	1.98E-25
ribonucleoside binding (GO:0032549)	3	1.98E-25
ribonucleotide binding (GO:0032553)	3	3.88E-25
ATT		
SUMO polymer binding (GO:0032184)	33	4.94E-02
SUMO binding (GO:0032183)	33	4.94E-02
ubiquitin-like protein binding (GO:0032182)	23	1.40E-03
calcium ion binding (GO:0005509)	8	1.81E-02
**Biological process**		
Vir		
ribonucleoprotein complex biogenesis (GO:0022613)	4	2.29E-03
Vir colon		
carbohydrate metabolic process (GO:0005975)	3	2.27E-02
ATT		
cellular protein catabolic process (GO:0044257)	4	3.48E-02
proteolysis involved in protein catabolic process (GO:0051603)	4	3.48E-02
protein catabolic process (GO:0030163)	4	3.68E-02

Were only analysed the annotated genes: Vir condition 191 genes, Vir Colon 304 genes and ATT 65 genes. The table present only the categories enriched 3 fold or more.
