# Supplementary material for: Extensive transcriptome analysis correlates the plasticity of Entamoeba histolytica pathogenesis to rapid phenotype changes depending on the environment
Source: Sci Rep. 2016 Oct 21;6:35852. doi: 10.1038/srep35852 (PMC5073345; doi:10.1038/srep35852)
Supplement: Supplementary Information [file srep35852-s1.pdf]

Extensive transcriptome analysis correlates the plasticity of *Entamoeba histolytica* pathogenesis to rapid phenotype changes depending on the environment.

Christian Weber [1,2], Mikael Koutero[3], Marie-Agnes Dillies[3,5] Hugo Varet[3,5], Cesar Lopez-Camarillo[4], Jean Yves Coppée[3], Chung-Chau Hon[1,2] and Nancy Guillén[1,2*]

1. Institut Pasteur, Cell Biology of Parasitism Unit, F-75015 Paris, France
2. Inserm, U786, F-75015 Paris, France
3. Institut Pasteur, Transcriptome and EpiGenome, BioMics, Center for Innovation and Technological Research, F-75015, Paris, France
4. Universidad Autonoma de la Ciudad de Mexico, Genomics Sciences Program, Mexico City, Mexico.
5. Institut Pasteur, Hub Bioinformatique et Biostatistique – Centre de Bioinformatique, Biostatistique et Biologie Intégrative (C3BI, USR 3756 IP CNRS) – F-75015 Paris, France

Supporting Information

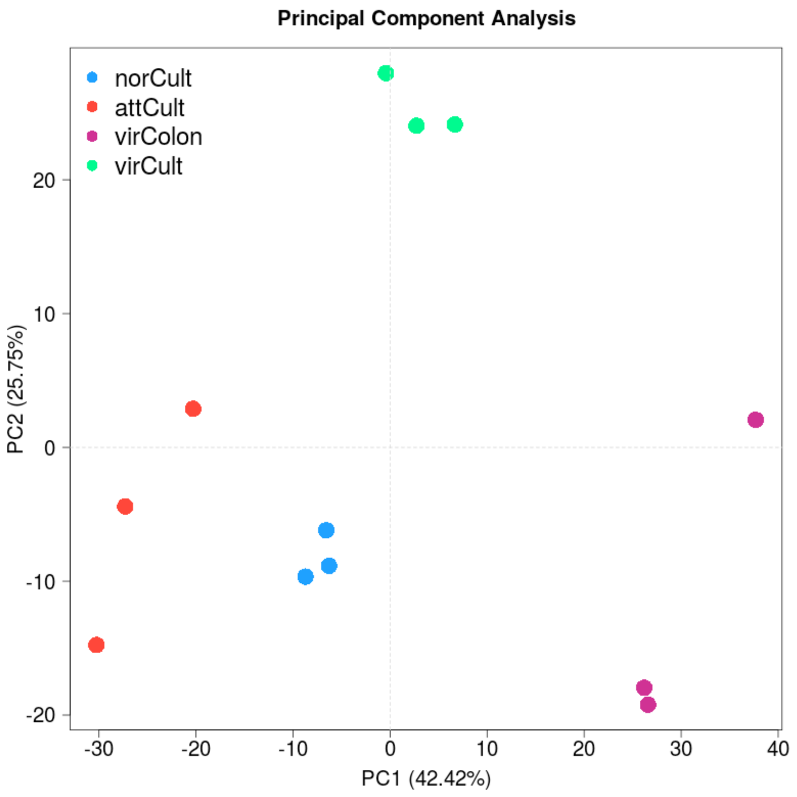

Figure S1. Principal component analysis. The graphs show the first factorial plan (axes 1 and 2) of the Principal Component Analysis (PCA) on the VST-transformed counts matrix. The four different biological conditions can easily be separated.

Scientific Reports. Data from Weber et al., 2016

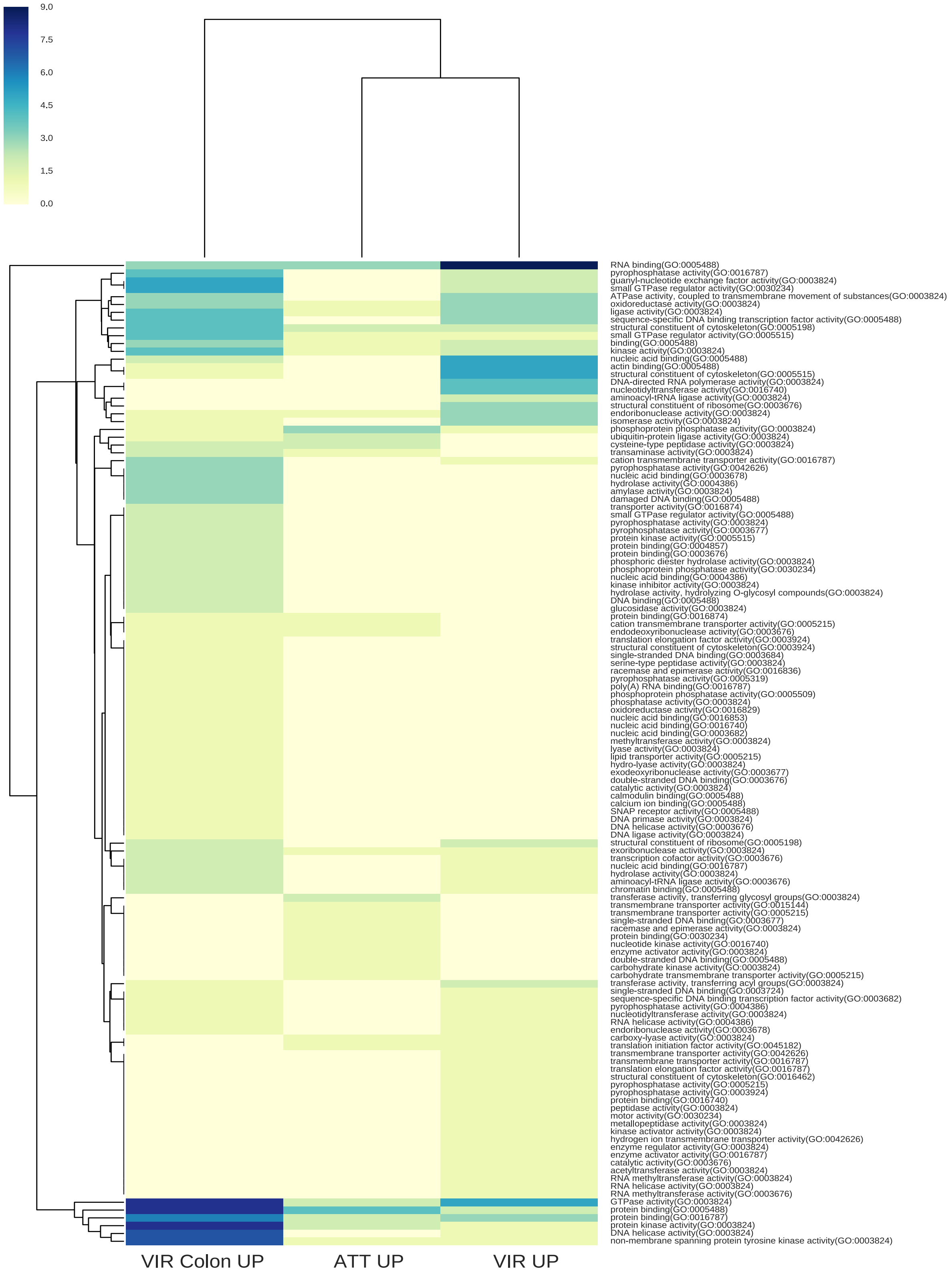

Figure S2. Molecular functions of genes uniquely upregulated in one given condition (VIR, VIR Colon, ATT) were obtained from PantherDB (www.pantherdb.org). The number of occurrences of each term was then counted in every conditions and plotted in a heatmap.

Supplemental Tables (14 in total)

1. Total transcripts displaying altered abundance in Vir (Table S1) VirColon (Table S2) and ATT (Table S3) conditions.

2. UP and down transcripts (cut off =3 or 0.3) from Vir (Table S4 and S5) VirColon (Table S6 and S7) and ATT (Table S8 and S9) conditions.

Table S10. Up regulated genes in common in microarrays and RNASEq approaches.

Table S11. Proteins from RNA metabolism class encoded by UP regulated genes in Vir condition

Table S12. Cytoskeletal proteins from UP regulated genes in Vir and Vir colon conditions

Table S13. Helicases and amylases class-UP regulated in VirCOLON condition

Table S14. Protein Degradation UP regulated in ATT conditions
